# Limpograss [*Hemarthria altissima*] Silage and Protein Supplementation as an Alternative Feed Option for Growing Heifers in North Florida

**DOI:** 10.3390/ani14162398

**Published:** 2024-08-19

**Authors:** Jose D. Pereira Neto, Jose C. B. Dubeux, Nicolas DiLorenzo, Igor L. Bretas, Mercia V. F. dos Santos, Martin Ruiz-Moreno, Priscila J. R. da Cruz, Erick R. da S. Santos, Luana M. D. Queiroz, Kenneth T. Oduor, Marcelo M. Vieira

**Affiliations:** 1 Department of Animal Sciences, Auburn University, Auburn, AL 36849, USA; jdp0130@auburn.edu; 2 North Florida Research and Education Center, University of Florida, Marianna, FL 32443, USA; ndilorenzo@ufl.edu (N.D.); ig.limabretas@ufl.edu (I.L.B.); ruizm001@ufl.edu (M.R.-M.); ldantasqueiroz@ufl.edu (L.M.D.Q.); kenneth.oduor@ufl.edu (K.T.O.); marcelomoretinvieira@gmail.com (M.M.V.); 3 Department of Animal Sciences, Universiade Federal Rural de Pernambuco, Recife, PE 52171-900, Brazil; mercia.vfsantos@ufrpe.br; 4 Department of Agronomy, Kansas State University, Manhattan, KS 66506, USA; pjrcruz@ksu.edu; 5 Department of Agriculture, Food and Nutritional Science, University of Alberta, Edmonton, AB T6G 2P5, Canada; ericksantos@ualberta.ca

**Keywords:** animal performance, conserving forage, forage intake, protein supplementation, weight gain

## Abstract

**Simple Summary:**

The fall gap represents a transitional phase between summer and winter that is characterized by feed scarcity due to the dormancy of warm-season forages while cool-season forages are not yet available. To address this challenge, various feed alternatives, such as hay, supplementation, stockpiling, and silage, are considered feasible options. Limpograss (*Hemarthria altissima*) is a unique warm-season forage because of its active growth in late fall and its slower decline in digestibility with increasing maturity compared to other grasses. Thus, limpograss silage can be an alternative to feed growing heifers in North Florida during the fall forage gap. A field trial was undertaken to assess the performance and nutritional responses of heifers fed ‘Gibtuck’ limpograss silage supplemented with protein. Twenty-four crossbred Angus × Brahman heifers were housed in a pen for the trial. Limpograss silage was provided without restriction to the heifers, while the supplement was offered at varying levels. The inclusion of protein supplementation alongside limpograss silage had a significant impact on animal dry matter intake, particularly affecting silage intake through a combination of substitutive and additive effects. Gibtuck limpograss silage associated with protein supplementation is an alternative to feed animals during forage-scarce periods in the southeastern United States.

**Abstract:**

Limpograss (*Hemarthria altissima*) is a warm-season perennial grass that has the potential to feed livestock during scarcity periods. This study evaluated the intake, nutrient digestibility, and animal performance of beef heifers fed ‘Gibtuck’ limpograss silage combined with different levels of a range cube supplementation. Twenty-four heifers (330 ± 16 kg live weight) were submitted to four different treatments with 6 replicates: (1) control, no supplementation + limpograss silage ad libitum; (2) 1.4 kg of supplement + limpograss silage ad libitum; (3) 2.8 kg of supplement + limpograss silage ad libitum; and (4) 4.2 kg of supplement + limpograss silage ad libitum. The apparent total tract digestibility of dry matter, organic matter, and crude protein showed a positive quadratic effect with increasing supplementation levels (*p* = 0.001, *p* = 0.002, and *p* < 0.0001, respectively). Overall, the supplement improved diet digestibility and total DM intake but reduced silage intake, indicating a combined effect (substitutive and additive effect) of the protein supplement. The increasing level of protein supplement increased the average daily gain with a quadratic effect (*p* ≤ 0.0001). Limpograss silage associated with supplementation can improve diet digestibility and increase the animal performance of growing heifers, providing an alternative for livestock in North Florida.

## 1. Introduction

Livestock systems in the southeastern United States primarily rely on perennial warm-season grasses, which are commonly planted in tropical and subtropical climate regions [1]. However, seasonality in forage production is a major challenge for grazing systems [2]. The production and nutritive value of these forages are limited during the winter, requiring supplementation to maintain adequate nutritional status for the animals [3]. Warm-season forages typically become dormant in late fall and winter due to shorter daylight hours, low temperatures, and frost [4], requiring alternatives to overcome this scenario. Most warm-season grasses have the potential to increase production during the growth period (summer), which is common in C4 plants. This is due to their photosynthetic system, which is associated with high efficiency in water and nitrogen use. Thus, conserving forage during periods of greater production for use in the winter appears to be a viable alternative [5]. In addition, conserved forage can be useful to feed animals during the fall gap between the dormancy processes of warm-season forages and cool-season forage growth for systems adopting cool-season forage overseeding.

Limpograss [*Hemarthria altissima* (Poir.) Stapf & Hubb.] is a warm-season perennial grass native to southern Africa and cultivated in tropical and subtropical regions [1]. This grass was introduced in Florida in 1964. The Floralta cultivar accounts for approximately 95% of the limpograss grown in Florida [6]. However, the limpograss hybrids cultivars ‘Kenhy’ and ‘Gibtuck’ were released in 2014 and have been recommended in Florida based on superior herbage accumulation, persistence under grazing defoliation at different locations, and greater performance under stockpiling when compared to Floralta or other hybrids [2,6].

Studies have reported on animals grazing stockpiled limpograss in subtropical regions [7,8,9]. Limpograss has a slower rate of digestibility, which declines with maturity, compared to other warm-season perennial grasses [10], with in vitro digestible organic matter (IVDOM) ranging from 520 to 594 g·kg^−1^ [2,6,10]. Due to its ability to initiate growth after a frost and its reasonable digestibility during the later stages of maturity, limpograss is considered a promising option for stockpiling [1]. Stockpiling limpograss in the fall seems practical, as the surplus forage produced during the summer months can be preserved for extended use. Using this material as silage could be useful to overcome the fodder shortage since silage is a feasible alternative to overcome the weather-related limitations of conserving warm-season grasses in Florida [5].

Limpograss has the potential to support livestock operations during periods when other warm-season perennial grasses would normally be dormant [10]. However, the low crude protein (CP) concentration may limit satisfactory animal performance [11,12,13]. When forage is available, and CP is a limiting factor, supplementation is necessary to increase diet CP and maintain or improve animal performance [14,15]. Limpograss usually has a low CP concentration, especially when stockpiled, with concentrations ranging from 34 to 125 g·kg^−1^ [2,5,6,8,15], requiring protein supplementation for beef cattle diets. In addition to low CP, limpograss has shown an approximately 26.5% undigestible protein fraction when harvested at 60 cm height and with a lag time of 21.4 h [13], which negatively affects the utilization of the B fraction of the protein compared to other grasses. These factors make limpograss a unique forage grass for tropical systems. Therefore, using limpograss silage could be a feasible option to bridge the forage shortage between seasons or during the cool season, provided a protein source is supplemented.

Several studies have evaluated the effects of CP supplementation on animal performance in forage-based systems [16,17,18,19,20]; however, there is a knowledge gap in terms of limpograss silage supplementation. The lack of studies can be explained by the recent spread of the limpograss cultivar ‘Gibtuck’ as an alternative for forage conservation and the difficulty of monitoring the variability in silage and supplement intake individually. Currently, the advent of precision livestock farming technologies allows monitoring of the individual and temporal variability of silage intake for greater accuracy in estimating supplemental nutrient requirements and animal performance. Automatic self-feeding systems have been used to monitor total diet intake but can affect the evaluation of forage intake by confounding the effects of the concentrate mixed with the forage. Therefore, the combined use of the GrowSafe^®^ (System Ltd., Airdrie, AB, Canada) feed bunk to monitor forage intake and the Super SmartFeed^TM^ (C-Lock Inc. Rapid City, SD, USA) to regulate the concentrate intake is an important contribution to determining suitable supplementation levels for beef cattle fed with limpograss silage. This is the first study combining precision technologies to evaluate animal performance with limpograss silage.

We hypothesized that feeding limpograss silage can be established as a useful alternative during the forage shortage between warm and cool seasons in the southeastern United States. Therefore, the objective of this study was to evaluate the effect of different levels of protein supplementation on the intake, digestibility, and animal performance of developing heifers fed a limpograss silage-based diet.

## 2. Materials and Methods

### 2.1. Experimental Design, Animals, and Treatments

This study was approved by the University of Florida Institutional Animal Care and Use Committee with the ID: IACUC202100000035. The experiment was performed in the Feed Efficiency Facility (FEF) at the North Florida Research and Education Center (NFREC) located in Marianna, FL (30°52′ N, 85°11′ W, 35 m asl) from April to July and lasted 63 days. Twenty-four crossbred Angus × Brahman heifers with an initial body weight (BW) of 330 ± 16 kg were used in this experiment. The experiment was conducted using a randomized complete block design, using the initial body weight of heifers as a blocking criterion. All heifers were housed together in a pen (22 m × 15 m) with a watering system. There were four treatments: (1) control, with no supplement and ad libitum access to Gibtuck limpograss silage; (2) 1.4 kg of cube supplement (32% CP and 68% TDN) in a dry matter (DM) basis and ad libitum access to Gibtuck limpograss silage; (3) 2.8 kg of cube supplement (DM basis) and ad libitum access to Gibtuck limpograss silage; and (4) 4.2 kg of cube supplement (DM basis) and ad libitum access to Gibtuck limpograss silage. Individual silage and supplement intake were monitored using automatic feeders. Thus, the animal was considered a replicate, totaling 6 replicates per treatment.

The Gibtuck limpograss used to make the silage was planted in early June 2022 at NFREC Marianna and fertilized with 67 kg N, 45 kg P, 90 kg K, and 16.8 kg S ha^−1^. After 12 weeks, in early September, the limpograss was harvested and chopped using a Claas JAGUAR 860 forage harvester (Claas, Harsewinkel, Germany) and then ensiled in a bag (3.65 m diameter) using a silage bagger Versa model ID1017 (Versa Internal Density^®^, Astoria, OR, USA). Limpograss samples were collected before the ensiling process for chemical analysis. The period between the ensiling and silo opening was eight months. In late-April 2022, the silo bag was opened, and the silage was offered daily to the heifers, twice a day, in the morning and the afternoon, for 63 d. The GrowSafe^®^ system (GrowSafe^®^ System Ltd., Airdrie, AB, Canada) was used to serve and record individual daily silage intake. The system was placed in a pen in the Feed Efficiency Facility of the NFREC. Particle size distribution of limpograss silage was determined using the Penn State Particle Separator (PSPS) technique. The PSPS consists of three sieves with fractions >19.0, >8.0, and <8.0 mm and a bottom pan. Three silage samples were collected from each feed bunk at different points right after the offer time. After the two rounds of shaking, the material from each sieve and the bottom pan was weighed and dried at 55 °C for 72 h to determine the DM concentration on each separator sieve [21].

A commercial range cube supplement with 32% CP was used in this trial as a protein supplementation obtained from a commercial plant in South Florida. The supplement was offered using the Super SmartFeed^TM^ (C-Lock Inc. Rapid City, SD, USA). This automatic self-feeding system was placed on the pen side with two trays available and set to offer the supplement every day after 0000 h since the system was restarted. Samples of cube and silage were sent to a commercial laboratory (Dairy One Forage Laboratory, Ithaca, NY, USA) for preliminary nutrient composition. The diet nutrient composition is presented in Table 1.

### 2.2. Intake and Average Daily Gain

All heifers had an electronic radio frequency identification (RFID) ear tag, identifying each animal in both automatic systems used in this trial. The GrowSafe^®^ system measured silage intake, and the Super SmartFeed^TM^ system measured supplement intake individually. The pen was equipped with three pairs of GrowSafe^®^ feed bunk to record intake by weight change, measured to the nearest gram. In this system, each animal with RFID had its feed behavior monitored, and the intake was recorded during the whole experiment. The silage was offered ad libitum, which means that orts were allowed, and every day before offering a new amount of silage, the orts were discarded. For the protein supplement, the Super SmartFeed^TM^ system offered the amount of supplement pre-determined for each treatment, computing the intake of each animal per day. The machine was filled up with supplements, and then the system released small portions (50 g every 10 s while the animal remained in the feeder bin) of the amount of supplement to each animal accessing the tray gate; this process prevented other animals that were not in the tray from consuming the supplement that was not for them. The animals’ live weights were assessed every 14 days throughout the experimental period. Fasting was not applied before weighing the animals. Rather, they were weighed over two consecutive days to obtain an average live weight [23]. To calculate the average daily gain (ADG), the difference between the final and initial body weight divided by the number of days of the animal performance trial was considered. In the whole trial, all the weights were recorded simultaneously, between 0800 h and 1000 h, before the silage offer time, to reduce the disturbance over animal feeding behavior. The gain-to-feed ratio (G:F) was calculated by dividing the ADG by the dry matter intake.

### 2.3. Apparent Total Tract Digestibility

At the beginning of the trial, there were five days of adaptation to facilities and feeding types of equipment, with all animals having access to the same amount of supplement in the SuperSmart Feed^®^ (1.4 kg·DM·d^−1^) and silage ad libitum in the bunk. After the adaptation period, all heifers were assigned to the diets. After 15 days, feed (silage and concentrate) and fecal samples were collected for five consecutive days to determine the apparent total tract digestibility of DM, OM, CP, and NDF. Feed samples were collected once daily in three different points from the feed bunk and the Super SmartFeed^TM^. The fecal samples were collected twice a day, at 8:00 a.m. and 4:00 p.m., directly from the rectal ampoule.

At sampling, all samples were placed in labeled plastic bags and stored at −20 °C until further processing. Samples were then thawed and dried at 55 °C until constant weight, ground in a Wiley mill Model 4 (Thomas-Wiley laboratory Mill, Thomas Scientific, Swedesboro, NJ, USA) to pass through a 2-mm screen, and pooled within each heifer for further determination of nutrient content, digestibility, and marker concentration. Indigestible NDF (iNDF) was used as an internal marker to obtain the apparent total tract digestibility of DM, OM, CP, and NDF calculated using Equation 1 [24,25]:

100 − 100 × [(marker concentration in feed/marker concentration in feces) × (nutrient concentration in feces/nutrient concentration in feed)](1)

### 2.4. Laboratory Analyses

To obtain the DM and OM values of feed and feces, approximately 1 g of sample was weighed in duplicate, dried in an oven at 100 °C for 24 h, and subsequently submitted at 550 °C for 6 h in a muffle furnace. To obtain the NDF concentration, approximately 0.5 g of feed and feces were weighed in duplicate inside F57 bags (Ankom Technology Corp., Macedon, NY, USA) and analyzed using heat-stable α-amylase and sodium sulfite [26] in an Ankom 200 Fiber Analyzer (Ankom Technology Corp.) and then acid detergent fiber (ADF). To obtain the concentrations of iNDF in feed and feces, samples were weighed (0.5 g) in Ankom F57 filter bags and then incubated (in situ) within the rumen of a cannulated steer for 288 h (12 d). After that, samples were washed and dried, and NDF analysis was performed as described by [24,25].

To obtain the CP concentration, feed samples were ball-milled in a Retsch Mixer Mill MM400 (Retsch, Haan, Germany) at 25 Hz for 9 min to reduce the particle size under 100 μm, and then approximately 5 mg was analyzed for total C and N with the Dumas dry combustion method using a CHNS analyzer vario MICRO Cube (Elementar, Frankfurt, Germany). Once the concentration of N was obtained, CP could be estimated by multiplying the total N concentration by a factor of 6.25.

### 2.5. Statistical Analysis

All the response variables were analyzed as a randomized complete block design. Animals in the treatments were considered random effects. Treatments were considered fixed effects. The nutrient intake, apparent total tract digestibility, and average daily gain variables were tested for linear and quadratic effects using orthogonal polynomial contrasts in the SAS PROC MIXED (SAS/STAT 15.1, SAS Institute). The ‘*lackfit*’ function was used prior to proceeding with additional order effects. Since the lack-of-fit test was not significant for all the quadratic models, cubic models were not tested. Significance was declared at *p* < 0.05, and tendencies were considered at 0.05 ≤ *p* < 0.10.

## 3. Results

### 3.1. Intake

**Figure 1:**
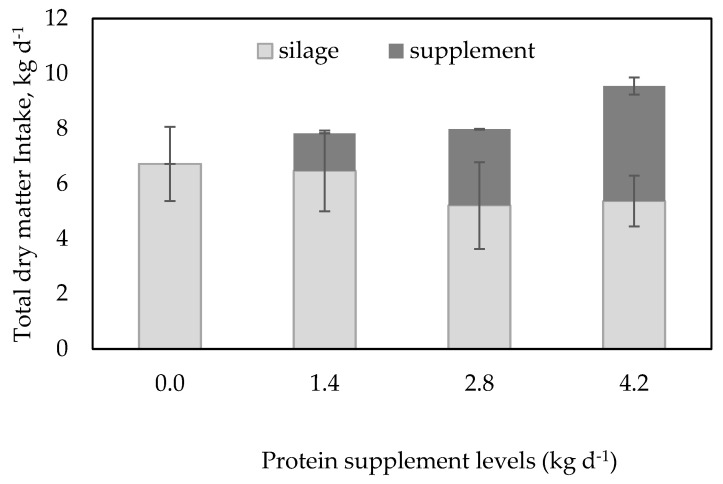
Total dry matter intake of growing Angus crossbred heifers fed limpograss silage and cubes in kg·d^−1^. 0 = Ad libitum access to limpograss silage. 1.4 = Ad libitum access to limpograss silage and 1.4 kg·DM·d^−1^ of range cube (32% CP, DM basis). 2.8 = Ad libitum access to limpograss silage and 2.8 kg·DM·d^−1^ range cube (32% CP, DM basis). 4.2 = Ad libitum access to limpograss silage and 4.2 kg·DM·d^−1^ of range cube (32% CP, DM basis). Limpograss silage intake had a linear effect (*p* = 0.02), SEM = 0.03; protein supplement intake had a quadratic effect (*p* < 0.01), SEM = 0.02.

There was a tendency for the inclusion of the range cube protein supplement to linearly (*p* = 0.07) decrease the limpograss silage dry matter intake (DMI; Table 2). As expected, the supplement DMI reached the set level for each treatment (1.4, 2.8, and 4.2 kg·day^−1^; *p* < 0.0001). Both total DMI (*p* = 0.01) and total DMI as %BW (*p* = 0.02) had a quadratic effect with the increase in supplement levels (Table 2). A combined substitutive and additive effect was observed with the inclusion of the supplement in total DMI (Figure 1).

### 3.2. Animal Performance

**Figure 2:**
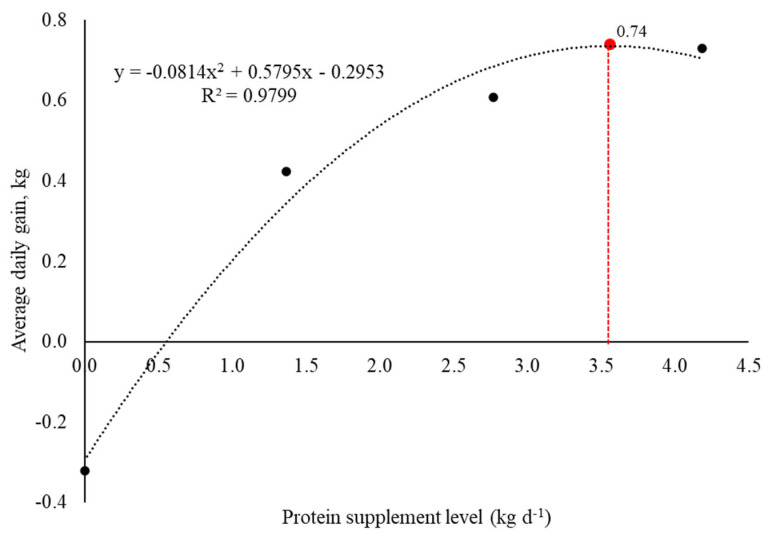
Relationship between protein supplement level (kg·d^−1^) and average daily gain (kg·d^−1^). The red dashed line represents the maximum value of average daily gain (0.74 kg) obtained with increasing supplement level and the respective supplement level (~3.6 kg·d^−1^).

Supplementing protein to a limpograss silage-based diet enhanced the ADG of growing heifers, showing a quadratic effect (*p* ≤ 0.0001; Table 2). Heifers fed only limpograss silage lost weight, while there was a quadratic increase in ADG from the first to the last level of supplement inclusion. The minimum supplementation level to ensure ADG = 0 (maintenance) was approximately 0.55 kg·day^−1^ of the protein supplement, while the maximum value of ADG obtained by the quadratic function was 0.74 kg with approximately 3.6 kg·day^−1^ of the protein supplement (Figure 2). There was a tendency for the gain-to-feed ratio to linearly increase with the treatments (*p* = 0.07).

### 3.3. Apparent Total Tract Digestibility

Dry matter, OM, and CP digestibility had a quadratic response (*p* = 0.001, *p* = 0.002, and *p* < 0.0001, respectively) with the inclusion of a supplement (Table 3). Including 1.4 and 2.8 kg of supplement increased the DM digestibility to 20 and 48 g·kg^−1^, respectively. The difference in DM digestibility between the treatment with only silage and the treatment with silage plus 4.2 kg of supplement was 64 g·kg^−1^. Crude protein digestibility significantly increased with the addition of the supplement, with a difference of 395 g·kg^−1^ compared to the treatment with only silage. However, neutral detergent fiber (NDF) digestibility decreased with the inclusion of the supplement and exhibited a quadratic effect (*p* < 0.0001).

**Figure 3:**
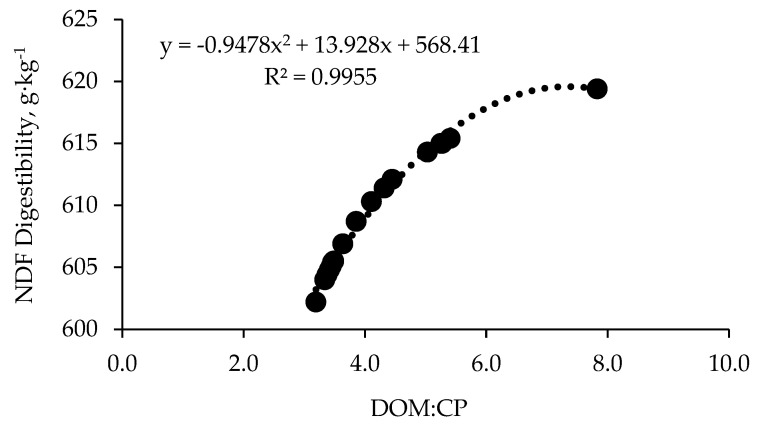
Relationship between diet degradable organic matter-crude protein ratio (DOM:CP) and neutral detergent fiber (NDF) digestibility.

There was a negative quadratic effect for NDF digestibility (*p* < 0.01) with the reduction of the ratio between digestible organic matter and CP (DOM:CP; Figure 3). The NDF digestibility decreased to 20 g·kg^−1^ with the inclusion of 4.2 kg of supplement in the diet, reaching a DOM:CP ratio of 3.4.

## 4. Discussion

### 4.1. Nutrient Intake

Protein supplementation can often enhance the intake and performance of animals fed low-quality forages [17,19,27]. Generally, adding protein affects forage intake, especially in low-quality forages with less than 7% of CP [28,29]. According to Sousa [19], protein supplementation can increase the dietary CP up to the minimum requirement (80 to 100 g·kg^−1^) to supply N for microbial growth and improve fiber degradation, which may increase forage intake. Supplementation enhances forage intake when the forage TDN:CP ratio exceeds 7 (indicating a nitrogen deficit relative to available energy). It also provides additional nutrients that can address the forage nutrient deficit, leading to increased total voluntary intake, digestibility, and animal performance [30,31,32]. Limpograss during late maturity has a low CP concentration, greater fraction C proportion, and longer lag time; thus, protein supplementation is recommended to meet the animal nutrient requirements [2,3,13,15]. Therefore, an improvement in voluntary forage intake was expected with protein supplementation. However, while total intake increased, silage intake decreased with higher levels of supplementation.

While there was a tendency to decrease silage intake, the increased total DMI can be associated with the associative effect of supplements on voluntary intake, digestibility, and energy concentration of diets. Supplements might improve diet quality by including energy and ruminal degradable protein (RDP), promoting fermentation, and increasing passage rate through the reticulum-rumen [33]. Voluntary intake is usually affected by multiple factors. Physiologic and physical mechanisms are major regulators of ruminant voluntary intake [34]. According to [30], the physiologic limitation occurs when animals meet their energy demand, while the physical restriction is associated with the fill-processing capacity of the animal. Thus, the higher diet passage rate through the reticulum-rumen in supplemented animals may have reduced the fill effect, allowing the supplemented heifers to increase their total intake. This statement is supported by the additive effect of protein supplement levels on total voluntary intake observed in our study. However, the intake will be limited by physiological or physical restriction at some point, which explains the quadratic effect observed for total voluntary DMI in our study. The physiologic regulation can also explain the decreasing silage DMI with increasing supplementation levels. It can be inferred that the substitute effect was likely happening on supplemented animals because the animals were reaching their energy requirement in advance and consuming less silage. The substitutive effect can either increase voluntary intake with improved forages or decrease voluntary intake when the supplement is provided with native species or straw [18]. Overall, the supplementation had a combined effect, incorporating both additive and substitutive effects [18,31], by reducing silage DMI and increasing total DMI. The substitutive effect on forage intake by increasing levels of protein supplementation was also observed by [35] in grazing steers. When the animals met their requirements, the authors observed decreased grazing and ruminating times, leading to decreased forage intake. In the current study, all heifers had access to the concentrate right after midnight, consuming the supplement before the silage was offered, which supports our explanation. However, substitution was not observed on a unit-by-unit basis, as the amount of supplement provided did not match the amount of silage not consumed.

Adams [36] attributed the reduction in voluntary forage intake to a forage DOM:CP ratio < 7, when diets are adequate in RDP. Reductions in forage intake with protein supplementation are expected in diets considered balanced in terms of RDP because animals can achieve their nutritional requirements faster through the supplement [18,36]. The observed depression in voluntary forage intake in our study may also be related to the DOM:CP ratio. Specifically, the DOM:CP ratio was 7.8 for non-supplemented diets (associated with greater forage dry matter intake), while it was less than 5 for supplemented diets (associated with lower forage dry matter intake). Typically, low-quality forages have a DOM:RDP ratio greater than 7, indicating an RDP deficit relative to available energy. Providing protein supplements can address this imbalance and may lead to increased total dry matter intake [18,36]. Some studies indicate that protein supplementation increases both passage rate and extent of digestion, leading to greater DMI when protein is supplemented [33]. In the current study, despite the tendency to increase DMI by increasing CP supplementation, there was a decrease in the NDF digestibility, indicative of a greater passage rate. However, the impact of supplements on forage intake is not uniform and can vary depending on the quality of the forage and the composition of the supplement [36].

It is important to note that the current trial employed a self-feeding system, which allowed for the accurate measurement of individual forage and supplement intake. This enabled precise assessment of total diet intake and digestibility, considering the interaction between forage and supplement in the rumen.

### 4.2. Animal Performance

The unsatisfactory animal performance of heifers fed only limpograss silage emphasizes that protein supplementation is necessary for ‘Gibtuck’ limpograss-based diets to increase animal performance [10]. The inclusion of range cube protein supplements improved the ADG of heifers fed a limpograss silage-based diet, supporting this statement.

The improved gain can be attributed to the availability of readily available protein in the rumen from the supplement, which enhanced diet digestibility. This resulted in a greater total DMI and higher levels of nutrients assimilated by the animals. However, ruminal energy utilization also should be considered when supplementing animals. The efficient utilization of volatile fatty acids (VFAs) requires an adequate supply of glucogenic substrates [33]. The gain between the levels 1.4 and 2.8 kg was considerable, with greater ADG at the higher level of supplementation. However, the quadratic pattern of increase in animal performance suggests that the higher supplementation level (4.2 kg·day^−1^) may not be economically and nutritionally viable for growing heifers. The level of supplementation of 2.8 kg·d^−1^ provides, on average, 290.7 g·CP·kg^−1^ DOM, which is close to 288 g·CP·kg^−1^ DOM indicated by [37] that maximizes intake, and there is no improvement in nitrogen utilization after this point. The surplus of nitrogen in relation to energy availability has several negative effects, including ATP deficiency in liver metabolism due to excessive utilization of the urea cycle, increased body heat production, and excess of ammonia in the blood that causes lower brain functioning due to energy deficits, leading to animal indisposition [37,38]. Economically, each additional unit of input yields only a small incremental output. Consequently, the effect of the second level of supplementation is less pronounced than the first, and subsequent levels will have even less impact on performance [28]. Indeed, our findings indicate that the maximum ADG of growing heifers obtained with the use of protein supplement would be achieved with an offer of approximately 3.6 kg animal^−1^ day^−1^ (32% CP and 68% TDN) of supplement, which is less than the higher level provided in this study. Our study also indicates that approximately 0.55 kg day^−1^ of that specific supplement would be enough to maintain growing heifers’ weight (ADG = 0) in a different scenario. The authors emphasize that it is essential to identify the point where cattle performance and cost expenses are optimized to define the optimum supplementation level. It is noticeable that the optimum level of growing heifers’ supplementation should consider the silage and concentrate costs for each location and the gain in reproductive performance or reduction in age at first calving for greater profitability.

As far as we are aware, this is the first study with different levels of limpograss silage supplementation. However, many trials were conducted in Florida to assess the animal performance with grazed, hay, or stockpiled limpograss with or without supplementation [3,8,11]. Newman [13] evaluated the ADG of beef heifers and sward characteristics on continuously stocked limpograss pastures grazed at different heights, with or without supplementation of 0 or 0.8 kg day^−1^ of 44% CP corn-urea meal. The study found that supplementation increased ADG by approximately 0.2 kg day^−1^ and that canopy heights did not affect the gains of supplemented heifers. However, there was a linear decrease in the extent of DM and CP degradation as canopy height increased.

Thus, using limpograss as an alternative to address periods of forage scarcity in beef cattle is a promising strategy. Limpograss is resilient during cool seasons and has the potential for high production and stable TDN concentration during late maturity, which supports animal gain [39]. The feed-to-gain ratio calculated for the diets did not show any statistical difference between treatments in our study. This means increasing animal performance by maintaining the same feed efficiency in diets based on limpograss silage is possible.

### 4.3. Apparent Total Tract Digestibility

Limpograss has a low CP concentration, particularly in late maturity. Additionally, since stems and leaves have different CP concentrations, changes in the leaf-to-stem ratio can significantly decrease the overall CP concentration of limpograss [1,39]. The CP concentration in our study was in the range reported by Vendramini and Moriel [1], which was approximately 73 g kg^−1^. However, the value obtained exceeded the value of 39 g kg^−1^ of CP reported by [14] for limpograss hay and the value of 40 g kg^−1^ of CP reported by Wallau [40] for stockpiled limpograss pastures but was lower than the value of 120 g kg^−1^ CP reported by Vendramini and Arthington [3]. The apparent total tract digestibility of OM in limpograss silage obtained in this study was 574 g kg^−1^, which is a reasonable value given that it was a warm-season perennial grass at an advanced stage of maturity. This result concurs with the statement by Vendramini and Moriel [1] that limpograss generally exhibits high digestibility following extended regrowth intervals. The IVDOM found in this current research was greater than the IVDOM found by Wallau et al. [40] (523 g kg^−1^), who evaluated the cultivar ‘Gibtuck’ in a stockpiling pasture at 12 weeks of age. Vendramini and Arthington [3] observed that the IVDOM of limpograss cultivar Floralta was 500 g kg^−1^. The limpograss used to make the silage of this study was harvested 84 days after staging.

The range cube protein supplement used in this current trial improved the digestibility of DM, OM, and CP in a limpograss silage-based diet. Other researchers also reported that the interaction between forage and supplements affects digestibility. Beaty [41], assessing the effect of protein concentration and frequency of supplementation of low-quality forages, found an increase from 487 to 545 g kg^−1^ for total DM digestibility in response to a protein supply that increased from 12 to 39% CP. Adams [36] reported an increase in DM digestibility of bermudagrass hay offered ad libitum with loose and extruded dried distillers’ grains cubes in heifers. The DM digestibility of bermudagrass alone was 400 g kg^−1^, while the total diet digestibility with a high level of protein supplementation was 550 g kg^−1^. Abreu [14] reported increased DM and OM digestibility in a diet based on limpograss hay supplemented with molasses (32% CP). Supplementation of 0.9 kg day^−1^ of molasses increased DM digestibility from 285 g kg^−1^ to 334 g kg^−1^ and OM digestibility from 346 g kg^−1^ to 389 g kg^−1^.

Several factors must be considered to explain the reported effects. Integrating different feeds into the rumen system can alter the rumen environment, which in turn affects the digestibility of feeds [42]. Low-quality forages typically have low concentrations of CP, which can reduce the efficiency of fiber-digesting microorganisms. The increased digestibility can be attributed to the additional protein available in the rumen and the supplementation strategy, which promoted microorganism growth and consequently improved digestibility [30]. The increase in DM, OM, and CP digestibility resulted from including an easily digested supplement. However, fiber digestion by ruminal microorganisms is a relatively slow process. The possible elevation in ruminal passage rate with increasing supplement levels likely reduced fiber degradation, explaining the reduction observed for NDF digestibility. A potential explanation not evaluated in the current study is the pH reduction caused by the interaction of supplements and forage in the rumen. Increasing concentrate levels in ruminant diets can lower rumen pH below 6.2 due to the rapid fermentation of carbohydrates by microorganisms [43]. Due to its rapid degradability, microorganisms may utilize more of the supplement for fermentation rather than digesting fiber. This can lead to the preferential fermentation of rapidly fermentable substrates, subsequently decreasing fiber digestibility [30]. This explanation is plausible since there was a strong relationship between the reduction in NDF digestibility and the DOM:CP ratio. Increasing the level of supplements in the limpograss silage diet shifted the DOM:CP ratio to 3.4 due to the higher diet CP. This elevation in supplementation improved digestion, enhanced the passage rate, and reduced fiber digestibility. Improved nutrient digestibility facilitates better nutrient assimilation to meet the animal nutritional requirements, enabling them to reach their productive potential. Also, increasing levels of supplements could elevate the starch concentration in the diet, which could be related to the reduction in fiber digestibility. Chase and Hibberd [44] reported a decrease in fiber digestibility of low-quality hay after increasing supplemental corn in the cattle diet.

It is worth noting that in addition to the low CP concentration of the diet containing only limpograss silage, there was a low CP digestibility (348 g·kg^−1^). The observed CP digestibility is comparable to 322 g·kg^−1^ reported by Lazzarini [45] using signal grass hay (*Urochloa decumbens* syn. Brachiaria). Underfeeding N below the threshold (60–80 g·CP·kg^−1^ DM) causes inefficient urea recycling that does not satisfy microorganism requirements, causing low digestibility and low intake [46]. When energy and protein are deficient, the animal can increase tissue mobilization to improve the N pool to sustain N recycling [47].

The silage fermentation parameters, with an adequate final pH (3.8), a high lactic acid concentration, and a negligible butyric acid concentration and particle size, suggest that the silage quality was not a limiting factor in our evaluations. This is a baseline study using Gibtuck limpograss silage and protein supplementation as an alternative to feed animals during forage-scarce periods or to conserve the excess of forage produced during the warm season in the southeastern United States. We emphasize that further studies are necessary to determine the optimal levels of CP supplementation for diets based on limpograss silage, considering factors such as animal category, sex, age, silage quality, CP sources, and local prices of beef and supplements.

## 5. Conclusions

Protein supplementation of growing heifers fed limpograss silage-based diets provides a combined effect, reducing voluntary silage intake but increasing total dry matter intake. Protein supplementation can improve the animal performance of growing heifers fed limpograss silage-based diets due to increased dry matter intake and apparent total tract digestibility of nutrients. The most feasible range cube protein supplementation level for growing heifers fed limpograss silage was 3.6 kg·d^−1^, resulting in an average daily gain of 0.74 kg. Studies assessing limpograss silage to feed beef cattle are scarce. Therefore, our findings are novel and indicate that limpograss silage can be an alternative to feed growing heifers when associated with protein supplementation. Further studies are required to extrapolate this feeding strategy to feed steers or mature cows in cow-calf operations in subtropical climates during the fall gap or cool season.

## Figures and Tables

**Table 1:** Chemical composition of limpograss silage and cube.

Item	Limpograss Silage	Cube
DM g·kg^−1^ DM	416	900
OM g·kg^−1^ DM	930	910
CP g·kg^−1^ DM	73	320
NDF g·kg^−1^ DM	674	301
TDN g·kg^−1^ DM	578 *	680
Calcium %	-	0.51
Phosphorus %	-	1.03
Magnesium %	-	0.43
Potassium %	-	1.16
Sodium %	-	0.120
Sulfur %	-	0.39
Lactic Acid, % DM ^1^	4.35	-
Acetic Acid, % DM ^1^	0.53	-
Lactic/Acetic Ratio ^1^	8.15	-
Propionic Acid, % DM ^1^	0.00	-
Butyric Acid, % DM ^1^	0.00	-
Isobutyric Acid, % DM ^1^	0.00	-
Total Acids, % DM ^1^	4.89	-
pH ^1^	3.80	-
NH_3_, CP Equivalent %, DM ^1^	0.26	-
NH_3_-N, % of Total N ^1^	3.61	-
VFA Score ^1^	8.32	-
PSPS Particle Size, % ^2^		
>19.0 mm	7.3	-
>8.0 mm	46.3	-
<8.0 mm	37.2	-
<4.0 mm	9.1	-

^1^ Dairy One Forage Laboratory. DM, dry matter; OM, organic matter; CP, crude protein; NDF, neutral detergent fiber, TDN, total digestible nutrients. ^2^ Penn State Separator. * Limpograss Silage TDN g kg^−1^ DM was obtained using the equation for forage (TDN = 0.889 − 0.00779 × ADF) as described by [22]. For silage, (*n* = 9) per bag. For cubes, (*n* = 3) per bag.

**Table 2:** Effects of different levels of range cube (32% crude protein) on the intake, average daily gain, and the gain-to-feed ratio of diets with limpograss silage ad libitum.

	Protein Supplement Levels (kg·d^−1^) *					Order Effect	
Intake, kg·d^−1^	0	1.4	2.8	4.2	SEM	*L*	*Q*
DM							
Silage	6.4	6.5	5.2	5.2	0.01	0.070	0.190
Supplement	-	1.4	2.8	4.2	0.01	<0.0001	<0.0001
Total	6.4	7.8	8.0	9.2	0.12	0.002	0.010
Total, %BW	1.9	2.3	2.3	2.7	0.03	0.010	0.020
OM Total, %BW	1.8	2.1	2.1	2.4	0.03	0.010	0.030
CP Total, %BW	0.1	0.3	0.4	0.5	0.02	<0.0001	<0.0001
NDF Total, %BW	1.3	1.6	1.6	1.8	0.02	0.010	0.020
ADG, kg	−0.32	0.42	0.61	0.73	0.03	0.0003	<0.0001
G:F	−0.05	0.05	0.08	0.08	0.005	0.070	0.100

* 0 = Ad libitum access to limpograss silage. 1.4 = Ad libitum access to limpograss silage and supplementation of 1.4 kg·DM·d^−1^ of the cube (32% CP, DM basis). 2.8 = Ad libitum access to limpograss silage and supplementation of 2.8 kg·DM·d^−1^ cube (32% CP, DM basis). 4.2 = Ad libitum access to limpograss silage and supplementation of 4.2 kg·DM·d^−1^ of the cube (32% CP, DM basis). SEM, Standard error of treatment means, *n* = 6 animals per treatment. L, linear effect; Q, quadratic effect; DM, dry matter; OM, organic matter; CP, crude protein; NDF, neutral detergent fiber; ADG, average daily gain; G:F, gain to feed. For silage and cubes samples, (*n* = 3).

**Table 3:** Effects of different levels of cubes (32% crude protein) on apparent total tract digestibility of heifers fed a limpograss silage-based diet and the DOM:CP ratio of the diet.

	Protein Supplement Levels (kg·d^−1^) ^1^					Order Effect	
Digestibility	0	1.4	2.8	4.2	SEM	*L*	*Q*
DM, g·kg^−1^	543	563	591	607	0.21	0.0002	0.001
OM, g·kg^−1^	574	593	620	638	0.23	0.0003	0.002
CP, g·kg^−1^	348	488	616	665	0.78	<0.0001	<0.0001
NDF, g·kg^−1^	620	614	608	604	0.01	<0.0001	<0.0001
DOM:CP	7.8	5.0	3.8	3.4	0.07	<0.010	<0.010

^1^ 0 = Ad libitum access to limpograss silage. 1.4 = Ad libitum access to limpograss silage and supplementation of 1.4 kg·DM·d^−1^ of the cube (32% CP, DM basis). 2.8 = Ad libitum access to limpograss silage and supplementation of 2.8 kg·DM·d^−1^ cube (32% CP, DM basis). 4.2 = Ad libitum access to limpograss silage and supplementation of 4.2 kg·DM·d^−1^ of the cube (32% CP, DM basis). SEM, standard error of treatment means, *n* = 6 animals per treatment. L, linear effect; Q, quadratic effect; DM, dry matter; OM, organic matter; CP, crude protein; NDF, neutral detergent fiber; DOM:CP, the ratio between organic matter digestibility and CP.

## Data Availability

The original contributions presented in the study are included in the article, further inquiries can be directed to the corresponding author/s.

## References

[1] Vendramini J., Moriel P. (2020). Management of Forages and Pastures in Lower-South: I-10 Corridor. Management Strategies for Sustainable Cattle Production in Southern Pastures.

[2] Wallau M.O., Sollenberger L.E., Vendramini J.M.B., Mullenix M.K., Quesenberry K.H., Gomide C.A.M., Costa e Silva V., DiLorenzo N. (2015). Herbage Accumulation and Nutritive Value of Limpograss Breeding Lines Under Stockpiling Management. Crop Sci..

[3] Vendramini J.M.B., Arthington J.D. (2010). Supplementation Strategies Effects on Performance of Beef Heifers Grazing Stockpiled Pastures. Agron. J..

[4] Blount A.R., Wallau M., Rios E., Vendramini J.M.B., Dubeux J.C.B., Babar A., Mulvaney M., Quesenberry K.H. (2020). 2020 Cool-Season Forage Variety Recommendations for Florida.

[5] Vendramini J.M.B., Desogan A.A., Silveira M.L.A., Sollenberger L.E., Queiroz O.C.M., Anderson W.F. (2010). Nutritive Value and Fermentation Parameters of Warm-Season Grass Silage1. Prof. Anim. Sci..

[6] Quesenberry K.H., Sollenberger L.E., Vendramini J.M.B., Wallau M.O., Blount A.R., Acuña C.A. (2018). Registration of ‘Kenhy’ and ‘Gibtuck’ Limpograss Hybrids. J. Plant Regist..

[7] Newman Y.C., Sollenberger L.E. (2005). Grazing Management and Nitrogen Fertilization Effects on Vaseygrass Persistence in Limpograss Pastures. Crop Sci..

[8] Sollenberger L.E., Ocumpaugh W.R., Euclides V.P.B., Moore J.E., Quesenberry K.H., Jones C.S. (1988). Animal Performance on Continuously Stocked ‘Pensacola’ Bahiagrass and ‘Floralta’ Limpograss Pastures. J. Prod. Agric..

[9] Vendramini J.M.B., Sollenberger L.E., De Oliveira F.C.L., Herling V.R., Gomes V.C., Sanchez J.M.D., Yarborough J.K. (2019). Herbage Characteristics of Continuously Stocked Limpograss Cultivars under Stockpiling Management. Crop Sci..

[10] Santos E.R.d.S., Dubeux J.C.B., Jaramillo D.M., Garcia L., Vendramini J., DiLorenzo N., Dantas Queiroz L.M., Pereira-Neto J.D., Sousa de Abreu D., Ruiz-Moreno M. (2022). Herbage Accumulation and Nutritive Value of Stockpiled Limpograss and ‘Tifton 85’ Bermudagrass. Crop Forage Turfgrass Manag..

[11] Da C. Lima G.F., Sollenberger L.E., Kunkle W.E., Moore J.E., Hammond A.C. (1999). Nitrogen Fertilization and Supplementation Effects on Performance of Beef Heifers Grazing Limpograss. Crop Sci..

[12] Holderbaum J.F., Sollenberger L.E., Moore J.E., Kunkle W.E., Bates D.B., Hammond A.C. (1991). Protein Supplementation of Steers Grazing Limpograss Pasture. J. Prod. Agric..

[13] Newman Y.C., Sollenberger L.E., Kunkle W.E., Chambliss C.G. (2002). Canopy Height and Nitrogen Supplementation Effects on Performance of Heifers Grazing Limpograss. Agron. J..

[14] Abreu D., Dubeux J.C.B., Queiroz L.D., Jaramillo D., Da Silva Santos E.R., Van Cleef F., Vela-Garcia C., DiLorenzo N., Ruiz-Moreno M. (2022). Supplementation of Molasses-Based Liquid Feed for Cattle Fed on Limpograss Hay. Animals.

[15] Aguiar A.D., Vendramini J.M.B., Arthington J.D., Sollenberger L.E., DiLorenzo N., Hersom M.J. (2015). Performance of Beef Cows and Calves Fed Different Sources of Rumen-Degradable Protein When Grazing Stockpiled Limpograss Pastures. J. Anim. Sci..

[16] Da Silva L.D., Pereira O.G., Da Silva T.C., Valadares Filho S.C., Ribeiro K.G. (2016). Effects of Silage Crop and Dietary Crude Protein Levels on Digestibility, Ruminal Fermentation, Nitrogen Use Efficiency, and Performance of Finishing Beef Cattle. Anim. Feed. Sci. Technol..

[17] Huuskonen A., Huhtanen P., Joki-Tokola E. (2014). Evaluation of Protein Supplementation for Growing Cattle Fed Grass Silage-Based Diets: A Meta-Analysis. Animal.

[18] Moore J.E., Brant M.H., Kunkle W.E., Hopkins D.I. (1999). Effects of Supplementation on Voluntary Forage Intake, Diet Digestibility, and Animal Performance. J. Anim. Sci..

[19] Sousa L.C.O., Palma M.N.N., Franco M.O., Detmann E. (2022). Does Frequency of Protein Supplementation Affect Performance of Cattle under Grazing in Tropical Pastures?. Anim. Feed Sci. Technol..

[20] Steen R.W.J. (1996). Effects of Protein Supplementation of Grass Silage on the Performance and Carcass Quality of Beef Cattle. J. Agric. Sci..

[21] Whitney T.R., Lee A.E., Williamson M.G., Swening C.D., Noland R.L. (2011). Use of the Penn State Particle Separator to Determine If Molasses Can Reduce Sorting of Ground Juniper When Juniper Is Used as a Feed Intake Limiter for Lambs. Anim. Feed Sci. Technol..

[22] Undersander D.J., Howard W.T., Shaver R.D. (1993). Milk per Acre Spreadsheet for Combining Yield and Quality into a Single Term. J. Prod. Agric..

[23] Warren H.E., Scollan N.D., Enser M., Hughes S.I., Richardson R.I., Wood J.D. (2008). Effects of Breed and a Concentrate or Grass Silage Diet on Beef Quality in Cattle of 3 Ages. I: Animal Performance, Carcass Quality and Muscle Fatty Acid Composition. Meat Sci..

[24] Cole N.A., McCuistion K., Greene L.W., McCollum F.T. (2011). Effects of Concentration and Source of Wet Distillers Grains on Digestibility of Steam-Flaked Corn-Based Diets Fed to Finishing Steers1. Prof. Anim. Sci..

[25] Krizsan S.J., Huhtanen P. (2013). Effect of Diet Composition and Incubation Time on Feed Indigestible Neutral Detergent Fiber Concentration in Dairy Cows. J. Dairy. Sci..

[26] Van Soest P.J., Robertson J.B., Lewis B.A. (1991). Methods for Dietary Fiber, Neutral Detergent Fiber, and Nonstarch Polysaccharides in Relation to Animal Nutrition. J. Dairy. Sci..

[27] Moore J.E., Kunkle W.E. Balancing Protein and Energy in Forages. Proceedings of the Florida Beef Cattle Short Course.

[28] Hersom M. (2007). Basic Nutrient Requirements of Beef Cows: AN190/AN190, 10/2007. EDIS.

[29] Moore J., Kunkle W., Brown W. Forage Quality and the Need for Protein and Energy Supplements. Proceedings of the Florida Beef Cattle Short Course.

[30] Mertens D.R., Grant R.J., Moore K.J., Collins M., Nelson C.J., Redfearn D.D. (2020). Digestibility and Intake. Forages.

[31] Moore J.E. (1980). Forage Crops. Crop Quality, Storage, and Utilization.

[32] Paterson J.A., Belyea R.L., Bowman J.P., Kerley M.S., Williams J.E. (1994). The impact of forage quality and supplementation regimen on ruminant animal intake and performance. Forage Quality, Evaluation, and Utilization.

[33] Mccollum F.T., Horn G.W. (1990). Protein Supplementation of Grazing Livestock: A Review1. Prof. Anim. Sci..

[34] Allison C.D. (1985). Factors Affecting Forage Intake by Range Ruminants: A Review. J. Range Manag..

[35] Mendes F.B.L., Silva R.R., De Carvalho G.G.P., Da Silva F.F., Lins T.O.J.D., Da Silva A.L.N., Macedo V., Abreu Filho G., De Souza S.O., Guimarães J.O. (2015). Ingestive Behavior of Grazing Steers Fed Increasing Levels of Concentrate Supplementation with Different Crude Protein Contents. Trop. Anim. Health Prod..

[36] Adams J.M., Robe J., Grigsby Z., Rathert-Williams A., Major M., Lalman D.L., Foote A.P., Tedeschi L.O., Beck P.A. (2022). Effects of Supplementation Rate of an Extruded Dried Distillers’ Grains Cube Fed to Growing Heifers on Voluntary Intake and Digestibility of Bermudagrass Hay. J. Anim. Sci..

[37] Detmann E., Valente É.E.L., Batista E.D., Huhtanen P. (2014). An Evaluation of the Performance and Efficiency of Nitrogen Utilization in Cattle Fed Tropical Grass Pastures with Supplementation. Livest. Sci..

[38] Poppi D.P., McLennan S.R. (1995). Protein and Energy Utilization by Ruminants at Pasture. J. Anim. Sci..

[39] Vendramini J.M.B. (2008). Use of Limpograss in Grazing Systems in Florida. FG.

[40] Wallau M.O., Vendramini J.M.B., Sollenberger L.E., Van Santen E., Aguiar A.D., Cunha O.F.R. (2020). In Situ Dry Matter and Crude Protein Disappearance Dynamics in Stockpiled Limpograss. Crop Sci..

[41] Beaty J.L., Cochran R.C., Lintzenich B.A., Vanzant E.S., Morrill J.L., Brandt R.T., Johnson D.E. (1994). Effect of Frequency of Supplementation and Protein Concentration in Supplements on Performance and Digestion Characteristics of Beef Cattle Consuming Low-Quality Forages. J. Anim. Sci..

[42] Doyle P.T., Francis S.A., Stockdale C.R. (2005). Associative Effects between Feeds When Concentrate Supplements Are Fed to Grazing Dairy Cows: A Review of Likely Impacts on Metabolisable Energy Supply. Aust. J. Agric. Res..

[43] Mould F.L., Ørskov E.R., Mann S.O. (1983). Associative Effects of Mixed Feeds. I. Effects of Type and Level of Supplementation and the Influence of the Rumen Fluid pH on Cellulolysis In Vivo and Dry Matter Digestion of Various Roughages. Anim. Feed Sci. Technol..

[44] Chase C.C., Hibberd C.A. (1987). Utilization of Low-Quality Native Grass Hay by Beef Cows Fed Increasing Quantities of Corn Grain. J. Anim. Sci..

[45] Lazzarini I., Detmann E., Sampaio C.B., Paulino M.F., Valadares Filho S.C., Souza M.A., Oliveira F.A. (2009). Dinâmicas de trânsito e degradação da fibra em detergente neutro em bovinos alimentados com forragem tropical de baixa qualidade e compostos nitrogenados. Arq. Bras. Med. Vet. Zootec..

[46] Sampaio C.B., Detmann E., Paulino M.F., Valadares Filho S.C., De Souza M.A., Lazzarini I., Rodrigues Paulino P.V., De Queiroz A.C. (2010). Intake and Digestibility in Cattle Fed Low-Quality Tropical Forage and Supplemented with Nitrogenous Compounds. Trop. Anim. Health Prod..

[47] Batista E.D., Detmann E., Titgemeyer E.C., Valadares Filho S.C., Valadares R.F.D., Prates L.L., Rennó L.N., Paulino M.F. (2016). Effects of Varying Ruminally Undegradable Protein Supplementation on Forage Digestion, Nitrogen Metabolism, and Urea Kinetics in Nellore Cattle Fed Low-Quality Tropical Forage1. J. Anim. Sci..

